# Privilege and burden of im‐/mobility governance: On the reinforcement of inequalities during a pandemic lockdown

**DOI:** 10.1111/gwao.12462

**Published:** 2020-06-14

**Authors:** Laura Dobusch, Katharina Kreissl

**Affiliations:** ^1^ Radboud Social Cultural Research Radboud University; ^2^ Department of Sociology University of Salzburg

**Keywords:** COVID‐19, inequality, interdependence, mobility, work

## Abstract

In order to contain the COVID‐19 pandemic, nation states have focused on the governance of im‐/mobilities: certain mobility restrictions have been enforced, while simultaneously some forms of mobility have been maintained or even enhanced in order to keep the system running in crisis mode. With a special focus on Austria, we analyse the specific politics of im‐/mobilities concerning the organization of paid work and show how the socio‐spatial conditions of who is permitted, denied or urged to work are inextricably linked to inequalities. It becomes apparent that while in principle all bodies are equally dependent on collective social relations and enduring infrastructure, not everybody contributes equally to their maintenance. In fact, the governance of im‐/mobilities follows and reinforces already prevalent inequality regimes based on class, gender and migration relations, thereby differentiating between bodies perceived as highly valuable and worth protecting and those categorized as less valued and potentially disposable.

## 1 INTRODUCTION

Nation states all over the world warn against tourist travel to any foreign destination. The International Monetary Fund (IMF) expects the ‘Great Lockdown’ during the coronavirus pandemic to lead to the ‘worst economic downturn since the Great Depression’ of the 1930s.^1^ Unemployment rates in Europe are assumed to double in the coming months and might only fall back to pre‐crisis levels by the end of 2021.^2^ The daily news coverage focuses on ‘coronavirus arithmetic’ and connected measures. Considering the omnipresence of the COVID‐19 outbreak, one could almost forget that the whole world switching simultaneously into crisis mode is not a ‘logical consequence’ to a ‘natural disaster’. Rather, also in this case, what is framed as a (global) crisis and which forms of crisis management are perceived as adequate are closely linked to prevalent power relations and societal inequality regimes.

While the situation of the refugees at the external borders of the European Union (EU) could also be described as an unignorable crisis that shakes the EU to its core, politics tend to individualize it as the result of the (wrong) choices of refugees themselves and externalize it as a problem of the neighbouring regions. The refugees’ fate *is untied* from that of EU citizens: the fact that ‘what gets crystallized at the margins of society … is embedded in a global social dynamic’ (Castel, 2003, p. xxii) is denied, simply because it is possible, because the relationship between refugees and the populations of the EU member states is characterized by fundamental asymmetries assigning the refugees a subject status as exposed supplicants without any bargaining power.

This works differently in the case of the COVID‐19 pandemic. Established national institutions do not focus on denying the constitutive interdependence underpinning human existence, but rather emphasize the mutual connectedness of their citizens. For instance, the coronavirus awareness campaign by the conservative‐dominated Austrian government propagates the slogan ‘Take care of yourself, take care of myself’ (‘Schau auf dich, schau auf mich’), thereby explicitly stating that the individual fate is inextricably bound to that of ‘the other’. And suddenly the notion of methodological individualism, which had instructed contemporary politics of nearly all political camps over the past few decades, is starting to show its cracks.

This acknowledgement of fundamental interdependency — which ‘is not the same as social harmony’ (Butler, 2015, p. 151) — is owed to specific characteristics of the COVID‐19 pandemic: first, the quality of the virus itself plays a significant role. Its high risk of infection combined with a long, asymptomatic incubation period makes people across all classes vulnerable to the virus:

*After all, the virus does not discriminate. We could say that it treats us equally, puts us equally at risk of falling ill, losing someone close, living in a world of imminent threat.* (Butler, 2020)

At least in the beginning, when the west largely dismissed China’s struggle with COVID‐19, the virus spread among members of the well‐connected, cosmopolitan elite as a global passenger, thus (also) carrying the health risk to gated communities and gentrified areas.^3^

Second, the mode of transmission, namely that *any* body can be a potential carrier of COVID‐19, made *any* interaction, *any* encounter between (groups of) people suddenly visible and meaningful both as life‐threatening and life‐sustaining. This was particularly the case for those interactions and encounters that had been, until then, taken for granted, such as undervalued and invisibilized infrastructural groundwork (e.g., care work, cleaning work, food supply). As the nation states’ crisis management in the form of lockdowns and severe restrictions of public life disrupted the seamless (re‐)production of basic living conditions, it became apparent that ‘the powerful and the secure are not placed on an Olympian plateau from which they can dispassionately contemplate the misery of the world’ (Castel, 2003, p. xxiii). Rather, an unintended consequence of the crisis management was to shed light on the fact that ‘everyone is dependent on social relations and enduring infrastructure in order to maintain a livable life’ (Butler, 2015, p. 21).

Third, and this is connected to the first two points, as the coronavirus can potentially infiltrate any social relationship and keeping in mind that a fundamental relationality is constitutive for the viability of human existence as such, the COVID‐19 crisis management cannot simply follow established strategies of externalizing costs (as happened in the financial crisis of 2007–2008) or blaming certain groups for ‘their own failure’ (as happened during the ‘long summer of migration’ of 2015). Instead, because anybody can transmit the virus, *everybody* needs to be taken into account when measures for the containment of the pandemic are designed.^4^

For national crisis management, this meant first and foremost introducing *governance of im‐/mobilities*: mobility restrictions such as closing national borders, avoiding contacts in public spaces, shutting down or decreasing public transport, home office incentives, etc. were enforced, while simultaneously, some forms of mobility were maintained (going to work in ‘system‐relevant’ jobs) or even enhanced (flying in care workers from Eastern Europe). By this specific governance of im‐/mobilities, it becomes apparent that while in principle all bodies are equally dependent on collective social relations and enduring infrastructure, not everybody equally contributes to their maintenance. In fact, the governance of im‐/mobilities follows and reinforces already prevalent inequality regimes based on class, gender and migration relations. Thereby it reveals a categorization into those whose lives are highly valued and preserved and those whose lives are less valued and potentially disposable (Bejan, 2020).

We illustrate this link between im‐/mobility governance and pre‐existing inequality regimes by the case of COVID‐19 measures of the Austrian government and how they affected the organization of paid work. The focus on paid work — *who is permitted, denied or urged to work under which im‐/mobility circumstances* — has several reasons. First, within the political discourse, all the COVID‐19 measures are discussed against balancing health needs and economic needs, which also implies ensuring that a certain amount of the population keeps working. Second, paid work is not only still a hugely significant enabler and indicator of one’s inclusion but also a necessity in order to cover the needs of daily life (Levitas, 1996). Third, how paid work is organized, its connected forms of rewards as well as the opportunity to work are fundamentally prestructured by class, gender and migration relations (Acker, 2006; Avent‐Holt & Tomaskovic‐Devey, 2019).

## 2 CRISIS MANAGEMENT AS IM‐/MOBILITY GOVERNANCE

The German writer Carolin Emcke describes the coronavirus pandemic as a ‘contrast medium’^5^ for social relations, a sort of magnifying glass for systemic cracks, societal vulnerabilities and inequalities. This does not merely affect the immediate impact of the disease and the distribution of health risks — who gets sick, who gets (good) treatment, who dies — but also the consequences of public health measures. It comes as no surprise that, when confronted with a hazard mainly characterized by unleashed mobility, ‘its restriction is justified as the key response’ (Düvell, 2020). Crisis governance targeting the containment of the virus mainly consists of *politics of im‐/mobility*, balancing the need between public health, maintaining the infrastructure of basic supplies and the demands of a capitalist economy.

These conflicting objectives considerably intersect when it comes to labour, leading to im‐/mobility enforcements that reveal and intensify immanent inequalities. While in certain professions employees are obliged to be physically present at their workplace in order to keep the system running, others (can) stay home. Further, national borders are reinforced and travel restrictions set in place — both legally and as a consequence of the shutdown of airports and traffic. Simultaneously, Eastern European migrant workers are flown in with charter planes for care or harvest work without proper health protection and pay. We argue that how governments have regulated ‘COVID‐19‐compatible’ socio‐spatial relations represents a crucial factor in this crisis and reveals constellations of inequalities in multiple ways, especially when it comes to the organization of paid work.

Mobility can be understood as a fundamental human right and affects a person’s opportunities to work, maintain social ties, get education, acquire basic supplies, etc. Therefore, it is deeply connected to social positioning, both shaping and being shaped by social relations and power asymmetries (Cook & Butz, 2018; Cresswell, 2010; Sheller, 2016). Formal and informal access regulations (e.g., borders, dress codes) allow or restrict people to move and participate differently in crucial areas of life, unequally distributing mobilities along inequality dimensions such as class, gender or migration. In this vein, scholars have coined the term *motility* to describe ‘the capacity of movement … under conditions of *one’s choosing*’ (Cook & Butz, 2016, p. 400; emphasis by authors), manifested in the form of a capital or a resource to gain access to economic, political, social and cultural opportunities (Elliot & Urry, 2010; Kaufmann, Bergman, & Joye, 2004). Motility as the potential to ‘access and appropriate the capacity for socio‐spatial mobility according to their circumstances’ (Kaufmann et al., 2004, p. 750) captures both privilege and social exclusion resulting from im‐/mobility in complex constellations as it enforces the focus on the option of choice to stay or be on the move. Motility is not only unequally distributed but also characterized by fundamental relationality: mobility and immobility are interrelated and occur ‘simultaneously among different social groups, creating complex and uneven mobility landscapes’ (Cook & Butz, 2018, p. 612). During the COVID‐19 pandemic, this relationally produced im‐/mobility landscape becomes particularly obvious: because some people are putting themselves at risk due to professionally required mobility, others can stay home. Motility, namely who can choose to stay home or move at their own will when it comes to paid work, is of crucial importance during the current crisis. In extreme cases, it can decide who lives or dies.

## 3 THE AUSTRIAN CASE

With the COVID‐19 legislation of 15 March 2020, the Austrian government introduced a set of measures aimed at containing the spread of the virus. This included the shutdown of sales and services except for basic necessities, such as supermarkets and pharmacies, and restricting individual mobility to going to work, necessary errands (e.g., groceries, medicine), helping other people and short walks in the fresh air. The latter was only legal alone or together with members of the same household, while all the activities required a minimum distance of one metre between individuals. Violations were monitored and sanctioned by the police, showing increased public presence. Employers were invited to facilitate home office whenever possible although this was not mandatory and therefore a question of goodwill. Evidently, this did not apply to employees working in so‐called 'system‐relevant' jobs such as shop assistants, pharmacists, health workers, staffers in public transport, etc. This is why, although schools and kindergartens officially closed down their educational operations, childcare support was still provided for parents working in these essential professions.^6^

### 3.1 Unequal immobilities: The blessing and curse of staying at home

When it comes to the consequences of COVID‐19 governance in terms of paid work, there are those denied going to their workplace and those permitted to stay home. For both forms of work‐related immobility, class matters significantly: first, the denial of going to work resulting in either unemployment or short‐time work,^7^ meaning the imposed reduction of work hours and income, disproportionally affects people with lower income and education levels. Although for three quarters of Austrian employees, work life has significantly changed since the beginning of the crisis (Prainsack, Kittel, Kritzinger, & Boomgaarden, 2020), people with compulsory education have by far the highest share of job losses (14.4 per cent) and short‐time work (39 per cent).^8^

Second, regarding the permission to stay home, a recent study shows striking inequalities when it comes to the distribution of motility (option of home office), both related to educational background and income (Pichler, Schmidt‐Dengler, & Zulehner, 2020). While a large number of people who graduated from high school (50.7 per cent) or university (63.4 per cent) work from home, six out of seven employees with compulsory education need to be physically present at their workplace. The higher the income, the larger the share of home office among employees.^9^ A recent analysis of phone companies’ mobility data draws a similar conclusion. In Vienna, Austria’s largest city and capital, mobility patterns quite accurately depict social stratification regarding regionalities: while in wealthier districts, people mostly stay home, mobility in working‐class neighbourhoods has not changed much since lockdown policies were implemented.^10^

However, this is not merely a result of working patterns, but also a consequence of housing. The lockdown and restrictions of movement exacerbate inequalities of living conditions connected to educational, socioeconomic and migration backgrounds. Larger families and residents of urban areas have less space available, children being particularly affected by cramped housing. In total, around 20 per cent of children living in Austria are confined to cramped housing conditions. Around 45 per cent of children with migrant background have little or no personal space versus 7 per cent of children with no recent ties to migration. Children from parents with compulsory education have a 51 per cent risk of living in cramped conditions versus 10 per cent from those with high school or higher education level (Bacher, 2020). This also explains why people in working‐class districts are — must be — more mobile. Temporary retreats from family and/or roommates as well as the need for fresh air are easier met in larger houses and a garden of one’s own. These inequalities are also reflected in the social phenomenon of correcting individuals who go outside. Particularly during the first weeks of mobility restrictions, social media was buzzing with public shaming of proclaimed negligent, unaware or even ‘stupid’ people who strolled around in parks before more and more voices pointed out the moral and classist undertone of a privileged perspective.

### 3.2 Urged mobilities: The necessity of leaving home

One of the first measures taken by states was the shutdown of national borders and the reinstallation of strict border controls, resulting in a halt in international travel for both business and leisure purposes. However, not for all. The Austrian care system, in particular 24‐hour care, substantially relies on migrant workers who usually take two‐ or four‐week turns between Austria and their home countries. While people living in Austria are encouraged to stay and the foreign ministry has engaged in bringing Austrian citizens back home, Eastern European migrant workers are flown in for care work with extra planes.^11^ At the end of March, 231 care workers — mostly women — from Romania and Bulgaria arrived in Austria and were subsequently put under quarantine without payment. Simultaneously, many colleagues have extended their stay due to travel restrictions and impending quarantine regulations when going back. Some of them have been working at their personal limit, up to eight weeks straight in an extremely demanding and underpaid profession without opportunities for retreat or seeing their loved ones, including family and children. Other carers are stuck in their home countries, cut off from income while still paying taxes and social security contributions into the Austrian system (Leichsenring, Staflinger, & Bauer, 2020a, 2020b).

This seems particularly appalling considering one of the latest changes in Austrian social policy, implemented by the then conservative right‐wing government: at the beginning of 2019, Austria introduced the indexation of family benefits and tax relief for children living in other EU member states according to the living standard of the state of residency. As a result, migrant workers from Eastern European countries, particularly women in care professions, suffer a considerable loss in their monthly earnings because those benefits used to bolster their wages. The European Commission has criticized this regulation’s discriminative nature because EU citizens working in Austria pay the same taxes as local employees.^12^

With the implementation of segmented mobility standards comes the classification of subjects into, as Raluca Bejan (2020) describes accurately, ‘those who deserve protection and those who do not’. The first are, in our case, the Austrian subjects, ‘whose lives and health are valued’ while the Eastern European workers are the ‘disposable subjects, those whose work matters more than their health, and whose health becomes vital only in relation to the domestic population’. This also becomes evident in recently published pictures of harvest workers waiting for their planes to Germany at the airport of Cluj/Napoca in Romania.^13^ Around 1800 people were queuing up and cramped together for hours without being able to follow physical distancing or hygiene regulations. However, on arrival in Germany, they were put under quarantine for 14 days in order to protect the domestic workforce. In a nutshell, during the coronavirus pandemic, certain groups of people were ‘mobilized’ while at the same time lacking motility.

## 4 CONCLUSION

As we have argued, the specific characteristics of the virus lay open both the life‐threatening and life‐sustaining nature of social relations underpinning our common viability. Through the sudden disruption of taken‐for‐granted encounters and interactions that have covertly kept our everyday lives going, the fundamental interdependence enabling any human existence becomes visible. However, interdependence should not be misunderstood as social harmony. In contrast, although *every*body’s being and becoming is dependent on and embedded in non‐/human relations, the maintenance of these relations is disproportionately shouldered by only *some* bodies. By outlining the reaction of the conservative‐dominated Austrian government to the coronavirus pandemic in the form of im‐/mobility politics, we could show that it is underpaid, undervalued and largely power‐distant groups of people — such as women care workers or migrant seasonal workers — that put their own lives at risk (by going out to work) in order to secure the other groups (who can stay home). We see that motility — the capacity to stay or move under conditions of one’s own choosing — is highly unequally distributed, connected to prevalent class, gender and migration relations.

Many current media commentaries and public voices praise the ‘new obviousness’ of who is carrying the burden of ‘system‐relevant work’ and express hope for a fairer financial and symbolic recognition of these professions post‐COVID‐19. However, we assume that this fundamental interdependence is not at all new for a globally operating economic elite, who is quite aware of its dependence on global value chains sustained through exploitative relationships. From our point of view, it is not the ‘epiphany’ of fundamental interdependence itself, but rather whether it is actually integrated into relationally oriented forms of (political) organizing ‘that are committed to fostering a sustainable interdependency on egalitarian terms’ (Butler, 2012, p. 149).

## Data Availability

The authors declared no potential conflicts of interests with respect to the authorship and/or publication of this article.

## ENDNOTES

^1^

https://www.imf.org/en/News/Articles/2020/03/23/pr2098-imf-managing-director-statement-following-a-g20-ministerial-call-on-the-coronavirus-emergency

^2^

https://www.reuters.com/article/us-health-coronavirus-eu-jobs/mckinsey-predicts-near-doubling-of-unemployment-in-europe-idUSKBN2210UZ

^3^
Austrian ski resorts, for example, were ‘coronavirus hotspots’ and led to the infection of middle‐class tourists who were able to afford an expensive vacation. However, the situation has changed: COVID‐19 now disproportionally hits the poor and the vulnerable such as black communities in the United States, immigrants in Sweden or favela residents in Brazil.

^4^
What happens if the crisis management ignores the fundamental relationality underpinning human existence is reflected in the case of Singapore: while being praised as a ‘crisis model student’ for its consequent contact tracing, Singapore was suddenly facing an unexpected increase of new infections related to migrant workers, who are living in cramped dormitories with poor sanitary conditions and who have been largely neglected by health authorities.

^5^

https://www.web24.news/u/2020/04/the-pandemic-is-an-authoritarian-temptation-that-invites-repression.html

^6^
This service was hardly used. Closing schools and kindergartens in combination with the discouragement of grandparents looking after their grandchildren has exacerbated already prevalent gender inequalities regarding the distribution of unpaid care work, suggesting that the pandemic will disproportionately disadvantage women and their (future) employment opportunities.

^7^
Short‐time work (Kurzarbeit) refers to a government programme aimed at the maintenance of jobs. Instead of lay‐offs, employers can reduce working hours to 10 per cent in three months. The pay gap is covered by the state, ultimately leaving employees with 80–90 per cent of their salaries.

^8^
In contrast, only 0.9 per cent of academics have lost their jobs, 17.9 per cent are subjected to short‐time work.

^9^
Inequalities regarding im‐/mobility when it comes to work also translate to the educational sector. Scholars draw attention to the potential widening of socioeconomic gaps among students due to lacking electronic equipment, housing conditions and parents’ learning support (Pichler et al., 2020). In short, homeschooling favours privileged kids.

^10^

https://www.wienerzeitung.at/nachrichten/chronik/wien/2058060-Klatscher-und-Beklatschte-in-der-Krise.html

^11^
In agriculture, there is a similar situation: regional institutions charter planes to fly in harvest workers from Romania.

^12^

https://www.derstandard.at/story/2000092969393/eu-sozialkommissarin-kritisiert-indexierung-bei-familienbeihilfe

^13^

https://www.tagesschau.de/ausland/rumaenien-erntehelfer-fluege-101.html
